# Differences in attentional function between experienced mindfulness meditators and non-meditators

**DOI:** 10.3389/fpsyt.2024.1341294

**Published:** 2024-03-14

**Authors:** Xiaohui Luo, Jia Zhao, Dongfang Zhao, Li Wang, Yi Hou, Yong Liu, Jing Zeng, Hong Yuan, Xu Lei

**Affiliations:** ^1^ Faculty of Psychology, Southwest University, Chongqing, China; ^2^ Peking University School of Public Health, Beijing, China

**Keywords:** mindfulness, meditation, attention, electroencephalography, P2, P3, delta, theta

## Abstract

**Introduction:**

Attentional enhancement has often been identified as the central cognitive mechanism underlying the benefits of mindfulness meditation. However, the extent to which this enhancement is observable in the neural processes underlying long-term meditation is unclear. This current study aimed to examine differences in attentional performance between meditators and controls (non-meditators) using a visual oddball task with concurrent electroencephalography (EEG) recordings.

**Methods:**

Thirty-four participants were recruited, including 16 meditators and 18 healthy controls, who were non-meditators. The participants completed a visual oddball task, using visual stimuli, and EEG recording.

**Results:**

Self-reports revealed that meditators had higher mindful attention scores than did the control group. The behavioral results showed that the meditators demonstrated faster reaction times than the non-meditators did. Neural findings indicated a higher P2 amplitude in the meditators than in the controls. The meditators demonstrated a significantly higher P3 in the target trials than in the distractor trials, which was not observed in the controls. Additionally, the time-frequency analysis demonstrated that the delta and theta powers in the meditators were significantly higher than those in the controls.

**Conclusions:**

The study suggests the meditators exhibited greater attentional performance than the controls did, as revealed by EEG and behavioral measures. This study extends previous research on the effects of mindfulness meditation on attention and adds to our understanding of the effects of long-term mindfulness meditation.

## 1 Introduction

Mindfulness meditation is a conscious, non-judgmental method of paying attention to the present state of affairs, state of consciousness, or mental process (1) and has been suggested as a tool to ameliorate various problems, including depression, anxiety, stress, insomnia, and addiction (1, 2). Attentional control is generally characterized as a central cognitive mechanism underlying the benefits of contemporary mindfulness meditation (3, 4). Although the ultimate aim of mindfulness interventions is not specifically to train attention, nurturing the skill of controlling attention is a fundamental aspect of developing and enhancing mindfulness (4, 5). Even individuals with no prior experience of meditation can improve their attentional functions with only a few minutes of mindfulness meditation practice or structured mindfulness training (6–10).

Long-term meditation can cause changes, including *state changes* that are dependent on meditation practice and *trait changes* that are independent of it. *State change* refers to the altered sensory, cognitive, and self-referential awareness that can arise during meditation practice, whereas *trait change* refers to lasting change in these dimensions that persist in the meditator irrespective of whether they are actively engaged in meditation or not (11). Many studies on the effects of meditation have focused on short-term changes in attentional neural plasticity by examining changes in the attentional features during or immediately before and after meditation (12–14). One study demonstrated that the impact of meditation on the attentional neural activity of experienced meditators was greater than that of non-meditators (14). However, it remains unclear whether the neuroplastic effects of long-term meditation practice can be observed in meditators during cognitive processing. Recent studies have shown that experienced mindfulness meditators exhibit superior performance on attention-related cognitive tasks, such as Go/No-go and global-to-local tasks, and heightened event-related potentials (ERPs), compared with non-meditators (15–17). However, one study found that meditators did not exhibit superior performance on oddball tasks (18), compared with non-meditators.

P2 and P3 are two attention-related markers observed in experienced meditators. P2 is primarily associated with early sensory processing during cognitive processes, particularly eliminating redundant information, reducing the allocation of attentional resources, and maintaining information processing efficiency (19). A study by Lutz, Slagter (20) found that meditators who practiced retreat meditation for 3 months showed an increase in P2 when attending to target tones during a dichotic listening task, whereas low-practice novice groups did not exhibit the same increase. P3 is commonly used as a cognitive indicator in healthy and clinical populations (21) and in meditation studies (17, 18, 22, 23). P3 reflects attentional engagement, memory updating, and information processing mechanisms and is often found in a variation of the visual or auditory oddball paradigm (24). Studies on experienced meditators have shown that P3 amplitudes increase in response to target stimuli (14, 25) and decrease in response to distracting stimuli (26). Additionally, after an 8-week focused attention meditation training, a basic meditation practice that cultivates attentional control and monitoring skills, participants exhibited an increase in P3 and a shorter reaction time (RT) during the three-stimulus oddball task than participants who received an 8-week relaxation training did (27). Although previous studies have suggested that P2 and P3 may be neural markers of attentional resource allocation during meditation and can be altered through meditation practice (14, 20, 25, 26), Payne, Baell (18) found no differences in P2 or P3 during an oddball task between meditators and non-meditators. This lack of difference may be due to the heterogeneity of meditation practices of the study participants. To explore this, this study attempted to observe P2 and P3 during non-meditative states, which can provide insights into the sustainable benefits of meditation on the attentional processes.

Theta (θ) and delta (δ) are two electroencephalography (EEG) oscillatory responses, which are altered by meditation (28, 29). Frontal midline θ activity is linked with concentrated attention engagement (30). Systematic reviews of EEG studies on meditation have consistently reported higher frontal midline θ activity during mindfulness meditation, and increases in θ activity have been linked with greater expertise in meditative practices (11, 22). Long-term meditators (14,240 h of meditation) robustly shifted states with enhanced θ power during meditation (compared with rest), whereas short-term meditators (1,095 h meditation) and non-meditators had not significantly changed (compared with rest), suggesting their restricted ability to shift between states (31). Therefore, some studies have recognized increasing θ power as a primary characteristic of meditation (29, 32). A recent study analyzing 10-min resting and 30-min meditative states in long-term meditators (> 5 years of meditation experience) found that the meditators shifted from rest to meditation with a gradual decrease in δ-band energy and a gradual increase in high-frequency band energy (28). Therefore, the decrease in low-frequency bands may also be one of the signs of entering a meditative state, implying that meditation may be a state of alertness (28). A distinguishing feature of many mindfulness-based interventions is integrating mindfulness into daily life. For example, mindfulness-based stress reduction (MBSR) emphasizes informal exercises that help practitioners incorporate mindfulness into their daily routines. This raises a fascinating research question: Can the meditative state be maintained during cognitive tasks outside of formal meditation practice, resulting in higher θ power and lower δ power and ultimately leading to superior cognitive performance in meditators compared with non-meditators?

Summarily, this study aimed to investigate whether idiosyncratic changes are present in attention-related neurophysiological markers (For example, P2 and P3) and meditation-related state markers (For example, θ and δ) in experienced mindfulness meditators in the absence of induced mindfulness states. To achieve this, the participants were required to perform an active visual oddball task, which is a paradigm for measuring attention-related neural activity (33). Furthermore, this study measured mindful attention, which is a central component of mindfulness meditation related to attention and awareness (34, 35), which assists individuals in recognizing and interrupting episodes of mental drift and self-focused thoughts (36, 37). We hypothesized that meditators would exhibit higher levels of mindful attention, better behavioral responses, and enhanced attention-related neural markers, compared with non-meditators. Specifically, we predicted that, compared with those with no experience of meditation, meditators would (1) report higher mindful attention scores; (2) show shorter RT and higher accuracy rate; (3) present higher power in P2, P3, and θ, and lower power in δ when presented with target stimuli.

## 2 Materials and methods

### 2.1 Participants

Thirty-four participants were recruited via online advertising, including 16 meditators (M ± SD_age_=44.13 ± 7.81; 13 females) and 18 healthy controls, which were non-meditators (M ± SD_age_ =40.61 ± 8.54; 12 females) (**Table 1**). The mediators were practitioners or advanced students of MBSR, which is one of the most widely recognized structured mindfulness-based interventions. Participants enrolled in the meditator group met the following inclusion criteria: (1) age of 18–65 years; (2) previous participation in an 8-week MBSR program; (3) at least 1 year of meditation experience; and (4) having practiced meditation for a minimum of 3 h per week over the last 3 months. This criterion is similar to considering an experienced meditator, as in previous studies (18, 20). Exclusion criteria included (1) a lifetime history of psychotic disorder, intellectual disability, organic medical disorders, bipolar disorder, posttraumatic stress disorder, or obsessive-compulsive disorder; (2) current alcohol or substance abuse or dependence; (3) significant suicidal ideation or behaviors; and (4) main practicing types of meditation different from those in the MBSR. The participants had a mean of 3.38 years of mindfulness meditation experience (1–9 years). Participants in the control group were included only if they had no experience with any form of meditation and had similar demographic information to the meditators. The participants were compensated with money (approximately 14 dollars) for their participation. All procedures performed in this study followed the ethical standards of the Declaration of Helsinki and were approved by the Institutional Review Board (IRB) of Southwest University of China (IRB code: H23115).

**Table 1:** The descriptive statistics, questionnaires, and behavioral data of the participants.

	Meditators (*N*)	Controls (*N*)	χ2
**Gender**			0.93
**Female**	13	12	
**Male**	3	6	
**Education degree**			0.04
**Bachelor’s degree**	1	1	
**Master’s degree**	12	14	
**Doctorate degree**	3	3	
	Meditators (M ± SD)	Controls (M ± SD)	*t*
**Age(years)**	45.25 ± 7.52	40.61 ± 8.54	1.67
**MAAS**	57.69 ± 7.95	39.56 ± 8.18	6.54***
**Reaction time(ms)**	491.90 ± 45.25	569.77 ± 80.52	-3.42**
**Accuracy(%)**	99.03 ± 0.03	99.84 ± 0.004	-1.24

M ± SD =Mean **±** standard deviation. ***p<0.001; **p<0.01.

### 2.2 Products and materials

The participants completed the task in a quiet room designed for EEG experiments. After providing informed consent, participants completed demographic information collection and self-report measurements of mindful attention. They then completed a behavioral task and EEG recordings.

Mindful Attention. The study used the Chinese version of the mindful attention awareness scale (MAAS) (38), which was originally developed by Brown and Ryan (35). The 15-item questionnaire was scored on a 6-point scale from 1 (almost always) to 6 (almost never), with higher scores indicating higher levels of mindful attention. The MAAS showed good reliability in this study (*α* = 0.92).

### 2.3 Behavioral task process and analysis

**Figure 1:**
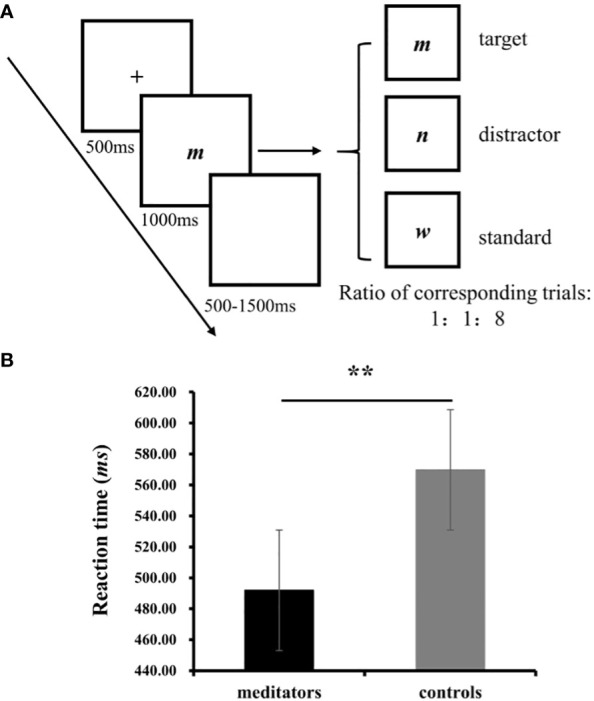
**(A)** One trials from the visual oddball task; **(B)** The results on RT from the oddball task. **p<0.01.

Participants completed a visual oddball task which used target, distractor, and standard visual stimuli (**Figure 1A**). They were instructed to respond to the target stimuli by pressing the “Enter” key on the keyboard, disregarding the other stimuli. The total task comprised three blocks with 120 trials per block, and the ratio of the target (a word “m”), distractor (a word “n”), and standard stimuli (a word “w”) was 1:1:8. A break was provided between blocks, where participants could choose whether to take a break or for how long. The task commenced after the participants understood the process; there was no practice block. It took approximately 30 min to complete the entire procedure, including the task. This was followed by a self-paced inter-trial interval. The experiment was programmed using E-Prime 2.0 (Psychology Software Tools, Pittsburgh, PA, United States), and the stimuli were presented on a Dell monitor (1,024 × 768 pixels), with each stimulus covering a visual angle of approximately 2.0° horizontal × 1.5° vertical.

Independent-sample t-tests were used to examine the accuracy rate and RTs. The accuracy rate denoted the ratio of correct responses (comprising responses indicating the correct recognition of target stimulus and non-responses to distractors and standard stimuli) to the total number of experimental trials, multiplied by 100%, whereas the response time was the average RT of the participants who correctly responded. All analyses were conducted using SPSS version 25.0 (IBM Corp., Armonk, NY). P-values were computed for deviations in all analyses based on the Greenhouse–Geisser method. *Post-hoc* t-tests were conducted, and the Bonferroni correction was applied for multiple pairwise comparisons.

### 2.4 EEG recording and analysis

Sixty-four-channel EEG was recorded while the participants performed the task (NeuSen. W64, Neuracle, Changzhou, China). Data were analyzed offline using MATLAB (The Mathworks, Natick, MA, 2019b) and preprocessed using EEGLAB (Delorme and Makeig, 2004). Data were epoched from -200 to 800 ms surrounding the stimulus onset for each trial. Trials containing electrooculogram artifacts (ocular movements and blinks), amplifier clipping, bursts of electromyographic activity, or peak-to-peak deflections exceeding ±80 µV were excluded from averaging before independent component analysis. This study focused on group differences in the responses to two types of rare stimuli: target and distractor. To analyze these differences, target and distractor data were obtained by subtracting standard stimulus data from the original ERP data for the targets and distractors. The analysis and results using the original data for the three stimuli, are is provided in the **Supplementary Material**.

Based on the topographic distribution of mean ERP activity, the mean ERP components and their respective time windows were identified as follows: P2 (245–365 ms) and P3 (435–585 ms). The following electrode sites were selected: Fz, FCz, Cz, and Pz. Furthermore, two (group: meditators and controls) × two (condition: target and distractor) × four (electrode site: Fz, FCz, Cz, and Pz) repeated-measure analyses of variance (ANOVAs) was conducted on the mean amplitudes of P2 and P3.

δ (2–4 Hz, 350–550 ms) and θ (4–8 Hz, 350–550 ms) brain activity was chosen for time-frequency analysis (the details methods of time-frequency see **Supplementary Material**). Two (group: meditators and controls) × two (condition: target and distractor) × four (electrode site: Fz, FCz, Cz, and Pz) repeated-measure ANOVAs were conducted on the values of δ and θ. All analyses were conducted via SPSS 25.0. Based on the Greenhouse–Geisser method, *p*-values were computed for deviation in all analyses. Simple effect analyses were performed for multiple pairwise comparisons.

### 2.5 Power analysis

A power analysis was performed to determine whether the sample size was adequate. With the sample size for analysis (N =34), α = 0.05 (two-sided), and a desired power of 80%, the smallest detectable between-group effect size calculated with G*Power 3.1.9.2 (39) would have been *d* = 0.32 for this study, which is compatible with the magnitude of the effects observed in previous related studies. In the study by Verhaeghen (5), the meta-analytic effect estimate of the comparison of the attention performance of long-term meditators and meditation-naive participants was Hedges’ *g* = 0.32.

## 3 Results

### 3.1 Demographic, self-reported and behavioral results

Meditators (M=45.25, SD=7.52) and controls (M=40.61, SD=8.54) did not differ significantly in age, t (1, 32) =1.67, p=0.104. The chi-square test revealed no significant differences in gender (χ2 = 0.93, p=0.34), and education levels (χ2 = 0.04, p=0.98). There was no significant difference between gender on self-reported and behavioral variables (see **Supplementary Table S1**).

The meditators reported significantly higher MAAS scores than the controls did (meditators: M=57.69, SD = 7.95; controls: M= 39.56, SD= 8.18, *t*(32) = 6.54, *p* < 0.001, *Cohen’s* =2.25). The results of the regression analyses showed that the longer individuals had been practicing mindfulness, the higher their MAAS (*β*=3.26, *p*<0.001) were likely to be.

No significant difference was observed between the groups in terms of accuracy (meditators: *M*=99.03%, *SD* = 0.01, controls: *M*= 99.84%, *SD*= 0.004, *t*(1, 32) =-1.24, *p* = 0.24). A significant difference was observed in RT, with the meditators responding more quickly, compared with the controls (meditators: *M*=491.90, *SD* = 45.25, controls: *M*= 569.77, *SD*= 80.52, *t* (1, 32) = -3.42, *p* = 0.002, *Cohen’s* =-1.17; **Figure 1B**).

### 3.2 ERP findings

**Figure 2:**
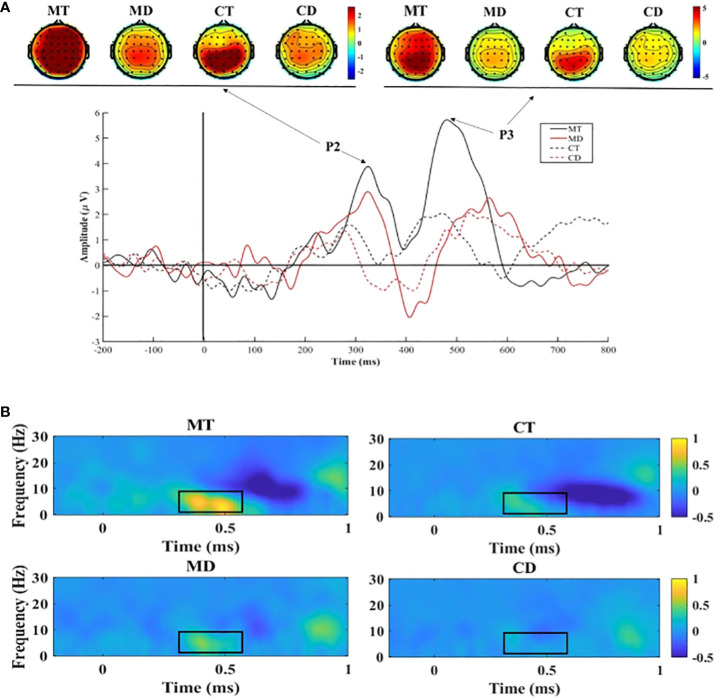
**(A)** Stimuli-locked, grand average waveforms of P2 and P3 at site Fz; **(B)** The delta and theta power at site Fz. MT, meditators’ response in target trials; MD, meditators’ response in distractor trials; CT, controls’ response in target trials; CD, controls’ response in distractor trials.

The grand average ERPs for P2 and P3 at Fz and the topography plots are shown in **Figure 2A**. The δ and θ powers at Fz are shown in **Figure 2B**.

#### 3.2.1 P2

The results for P2 showed a significant main effect of group (*F* (1,32)=12.11, *p*=0.001, ƞ^2 ^= 0.921). The mean P2 amplitudes in the meditator group (*M*=1.63, *SD*=0.29) were significantly higher than those in the control group (*M*=0.24, *SD*=0.27). The results also showed an interaction between the group and electrode site (*F*(1,32)=4.277, *p*=0.047, *η* ^2^ = 0.518). The simple effect analysis showed that the P2 of meditators was significantly higher than that of the controls at Fz (*F* [1, 32]=10.75, *p*=0.003, η^2^ = 0.89), FCz (*F* [1, 32]=12.89, *p*=0.001, η2 = 0.94), and Cz (*F* [1, 32]=14.70, *p*<0.001, η^2^ = 0.96).

#### 3.2.2 P3

No significant group difference was observed in the results for P3 amplitudes (*F*(1,32)=1.05, *p*=0.31, ƞ2 = 0.17); however, the follow-up simple effects analysis between group and condition found that the meditators had higher P3 amplitudes (*M*=4.54, *SD*=1.14) under target trials than under the distractor trials (*M*=2.48, *SD*=1.07; *F* (1,32)=14.24, *p*<0.001, ƞ2 = 0.96), which was not observed in the controls.

#### 3.2.3 δ

The results on δ showed a significant main effect of group (*F* (1, 32)=6.86, *p*=0.013, η^2^ = 0.72), and the meditators (*M*=0.33, *SD*=0.06) had a significantly higher δ power than the controls did (*M*=0.10, *SD*=0.06). The results also showed an interaction between the group and electrode site (*F*(1, 32)*=*8.33*, p=*0.007, η^2^ = 0.80). The simple effect analysis showed that δ power of the meditators was significantly higher than that of the controls at Fz (*F* [1, 32]=8.10, *p*=0.008, η^2^ = 0.79), FCz (*F* [1, 32]=7.71, *p*=0.009, η^2^ = 0.77), and Cz (*F* [1, 32]=5.60, *p*=0.024, η^2^ = 0.63). Additionally, an interaction between group and condition showed that the meditators had higher δ power (*M*=0.46, *SD*=0.10) under target trials than under the distractor trials (*M*=0.14, *SD*=0.10; *F*(1, 32)=8.54, *p*=0.006, ƞ^2^ = 0.81), which was not observed in the controls.

#### 3.2.4 θ

The results on θ showed a main effect of group (*F*(1, 32)=7.46, *p*=0.01, η^2^ = 0.75), and the θ power of the meditators (*M*=0.31, *SD*=0.07) was significantly higher than that of the controls (*M*=0.04, *SD*=0.07). The results also showed an interaction between the group and electrode site (*F* (1, 32)=8.44, *p*=0.007, η^2^ = 0.80). The simple effect analysis showed that the θ power of the meditators was significantly higher than that of the controls at Fz (*F* [1, 32]=9.08, *p*=0.005, η^2^ = 0.83), FCz (*F* [1, 32]=9.48, *p*=0.004, η^2^ = 0.85), and Cz (*F* [1, 32]=6.67, *p*=0.015, η^2^ = 0.71). Additionally, an interaction between group and condition showed that the meditators had a higher θ power (*M*=0.40, *SD*=0.10) under target trials than under distractor trials (*M*=0.05, *SD*=0.09; *F* (1, 32)=5.91, *p*=0.02, η^2^ = 0.66), which was not observed in the controls.

The results of other analyses unrelated to the group are presented in **Supplementary Table S3**.

## 4 Discussion

This study used a visual oddball task, which is considered a measure of neural activity related to attention (33), to examine differences between meditators and non-meditators. The examined differences included mindful attention (as measured by the MAAS), behavioral indices (RT and accuracy) in a cognitive task, attention-related ERPs (P2 and P3), and neurophysiological indices related to the meditative state (δ and θ), all of which were assessed in the absence of induced mindfulness. Meditators reported higher levels of mindful attention, and showed faster RTs and higher P2 amplitudes, δ and θ power than controls did. The meditators showed significantly higher P3 amplitudes in target trials than in distractor trials, whereas the controls did not.

The current results confirmed previous findings by demonstrating that meditators have better cognitive performance than non-meditators do. Specifically, meditators had faster RTs, compared with non-meditators. Research has shown that meditation can improve sustained attention (7, 9). Additionally, meditation affects selective attention allocation by enabling efficient allocation of attentional resources, expanding attention, speeding up the recognition of target stimuli, and improving attentional switching (12, 15, 40, 41). These results suggest that meditation may be a valuable tool for improving cognitive function.

This study showed larger P2 amplitudes in meditators than in controls in all conditions. Depending on the stimuli and tasks presented, P2 amplitudes are related to the filter mechanisms involved in attention allocation (42). Generally, it is modulated by attention and associated with the initiation of an executive process when identifying unusual stimuli (20, 42). The P2 is an indicator of attentional engagement during cognitive processing (43). In our study, meditators showed higher P2 amplitudes and shorter RTs, suggesting more efficient attentional functioning during stimulus perception, although the task accuracy rate was approximately the same for both groups (potentially due to a ceiling effect).

The P3 results indicate that the meditators showed a stronger neural response to the target stimulus than the distractor stimulus, whereas the controls showed no such difference. Regarding working memory, the P3 signal may arise from the initial need to increase target attention during stimulus detection (24); therefore, if P2 is related to an individual’s ability to detect stimuli, P3 is more related to stimulus recognition. Meditation was associated with higher P3 amplitudes in most tasks requiring attention to task-relevant stimuli (14). Meditators show stronger neural responses to target stimuli, suggesting more efficient attentional functioning, enabling better detection of targets and distractors.

Consistent with previous related research, the sequential appearance of P2 and P3 reflects the meditators’ ability to quickly identify and discard distracting stimuli. This finding may be related to the fact that the practice of meditation fosters *background awareness* (44), which is the capacity of individuals to maintain their attention to contextual information while their primary focus is on a given object. Individuals with higher *background awareness* are more likely to perceive a sense of conflict between the goal and context during task performance. The higher the sense of conflict, the more likely it is for introspection to occur and make it easier to discriminate between stimuli, resulting in mind-wandering reduction (7). Due to their greater depth of processing, meditators may be able to selectively attend to either local or holistic information based on the requirements of the task than non-meditators, who may have more limited attentional control (15). Specifically, in this study, during the simpler oddball task, experienced meditators, even when attending efficiently to rare stimuli (higher P2 amplitude for both target and distractor stimuli), were able to detect conflicts with the target and distractor stimuli (higher P3 amplitude for the target stimuli). Furthermore, this study’s results differ from those of Payne, Baell (18). The meditators in this study, who were MBSR course practitioners or advanced students, were uniform in their type of meditation practice, particularly in their core attitudes, thus reducing heterogeneity within the meditation group. This reminds us of the need to explore the unique neurophysiological mechanisms associated with different types of meditation.

The δ wave is widely regarded as a characteristic of slow-wave sleep and is typically associated with a state of deep relaxation (45). Previous studies have shown that meditators have higher δ activity in a non-meditative resting state than controls do (28, 46, 47). δ activity has been linked with cognitive processes, predominantly attentional processes (48). For example, Ishii, Canuet (49) concluded that frontocentral-parietal δ synchronization is functionally involved in auditory attention by recording magnetic responses during an auditory oddball task in 12 healthy participants. Therefore, higher δ power may indicate lower cognitive effort and better cognitive function in cognitive tasks. θ activity plays a vital role in the physiological mechanisms underlying cognitive control (50). Increased θ activity is associated with better cognitive control (30). Previous research has shown that higher δ- and θ-band activity is associated with response inhibition processes in attentional tasks, as evidenced by higher inhibitory responses to distractor stimuli (51). Collectively, the higher δ and θ powers indicate that meditators were potentially in a relaxed yet cognitively robust state whilst undertaking the task.

The results on δ activity were inconsistent with the hypothesis, reflecting higher δ power. This may be because the hypothesis was based on the results of previous studies related to meditators in the meditative state and not engaged in performing cognitive tasks. Most mindfulness meditation needs practitioners to control attention or direct it to non-explicit objects (4), which requires more attentional resources, leading to lower δ power in the meditative state compared with the resting state (28). The accuracy results for meditators (*M*=99.03%) indicated that the difficulty of the task used in this study may be lower than that of meditation.

This study had some limitations. First, no significant difference was observed between the two groups regarding task accuracy in the experiment. Some participants demonstrated exceptional performance, which may indicate a ceiling effect due to the oversimplified task. Further research could investigate the differences in attentional performance between meditators and controls with limited or abundant attentional resources by varying the level of difficulty of the experiment. Second, this study only assessed mindfulness and attention in one dimension. Future research may consider other characteristics (such as describing, non-reactivity, and non-judgement) or measure mindfulness as a multidimensional construct (52), particularly given criticisms of the MAAS (53). Third, the results of power analysis suggested that the present study had the power to detect effect sizes by comparing with the previous study (5). However, the limitation of sample size in this current study was still noted, and it is necessary to explore the effect of long-term mindfulness meditation in a large meditator sample in future study. Finally, this study focused on temporal information suggested by high temporal resolution EEG data and did not address spatial information regarding brain activity. Future studies should investigate the temporal and spatial neural metrics in the oddball task by collecting both EEG and functional magnetic resonance imaging data.

Summarily, compared with meditation-naïve individuals, experienced mindfulness meditators exhibited higher levels of mindful attention and elevated neurophysiological activity in attention-related markers (P2 and P3) and state-related markers (θ and δ) during a non-meditative state. This study extends previous research on the effects of mindfulness meditation on attention and adds to our understanding of the effects of long-term mindfulness meditation.

## Data availability statement

The datasets presented in this study can be found in online repositories. The names of the repository/repositories and accession number(s) can be found below: https://www.scidb.cn/en/detail?dataSetId=68c96e2326ba415987afacaadf0767d8.

## Ethics statement

The studies involving humans were approved by The Institutional Review Board (IRB) of the Southwest University of China. The studies were conducted in accordance with the local legislation and institutional requirements. The participants provided their written informed consent to participate in this study.

## Author contributions

XiL: Conceptualization, Formal analysis, Investigation, Methodology, Writing – original draft, Writing – review & editing. JZh: Formal analysis, Methodology, Writing – review & editing. DZ: Conceptualization, Methodology, Writing – review & editing. LW: Investigation, Writing – review & editing. YH: Investigation, Writing – review & editing. JZe: Resources, Writing – review & editing. YL: Writing – review & editing. HY: Conceptualization, Methodology, Writing – review & editing, Funding acquisition, Project administration. XuL: Funding acquisition, Writing – review & editing.
