# Supplementary material for: Differences in attentional function between experienced mindfulness meditators and non-meditators
Source: Front Psychiatry. 2024 Mar 14;15:1341294. doi: 10.3389/fpsyt.2024.1341294 (PMC10982879; doi:10.3389/fpsyt.2024.1341294)
Supplement: Supplementary file 1 [file DataSheet_1.docx]

Supplementary Material

# The methods of time-frequency analysis

The EEG data was subjected to time-frequency analysis using a windowed Fourier transform (WFT) featuring a Hanning window of fixed width (250 ms). The WFT provided a time-frequency spectral estimate F(t,f) that was complex at every point (t,f) of the time-frequency plane, ranging from −200 ms to 800 ms in the time domain and from 1 Hz to 40 Hz (in steps of 1 Hz) in the frequency domain, for each single trial. A baseline correction was performed at the subject level by utilizing the pre-stimulus interval (pre-stimulus -200 to 0 ms) to derive the power change, which was computed utilizing the following formula:

TFD (t, f) = P (t, f) – R (f)

where P(t, f) = |F(t, f)|2 represents the power spectral density at a particular time-frequency point (t, f), and R(f) denotes the average power spectral density of the signal enclosed within the pre-stimulus reference interval (−200 to 0 ms before the onset of the stimulation) for each estimated frequency f.

# Results between Gender

| Table S1. The results of independent t analysis between gender | | | | | |
| --- | --- | --- | --- | --- | --- |
| **Variance** | **Female (M±SD)** | **Male (M±SD)** | ***t*** | ***Sig.*** | ***Cohen's*** |
| Age（years） | 43.88±8.705 | 39.78±6.515 | -1.285 | 0.208 | -0.5 |
| MAAS | 49.04±11.137 | 45.44±15.026 | -0.757 | 0.455 | -0.294 |
| Reaction time（ms） | 530.19±84.21 | 541.29±51.59 | 0.369 | 0.714 | 0.14 |
| Accuracy（%） | 0.993±0.02 | 0.998±0.002 | 0.71 | 0.483 | 0.276 |
| **Note.** MAAS=the Mindful Attention Awareness Scale | | | | | |

# The results of the repeated measures ANOVAs using three stimuli original data

## EEG Recording and Analyses

Sixty-four-channel EEG was recorded while the participants performed the task (Brain Products GmbH, Gilching, Germany). Data were analyzed offline using MATLAB (The Mathworks, Natick, MA, 2019b) and preprocessed using EEGLAB (Delorme and Makeig, 2004). Data were epoched from -200 to 800 ms surrounding the stimulus onset for each trial. Trials containing electrooculogram artifacts (ocular movements and blinks), amplifier clipping, bursts of electromyographic activity, or peak-to-peak deflections exceeding ±80 µV were excluded from averaging before independent component analysis.

Based on the topographic distribution of the mean ERP activities, the mean of ERP components and their respective time windows were identified as follows: P2 (160–280 ms), and P3 (260–380 ms). The following electrode sites were selected, Fz, FCz, Cz, and Pz. 2 (group: meditators and controls) × 3 (condition: standard, target and distractor) × 4 (electrode site: Fz, FCz, Cz, and Pz) repeated measures ANOVAs were conducted on the mean amplitudes of P2 and P3 respectively.

Delta (δ, 2-4 Hz, 250-450 ms) and theta (θ, 4-8 Hz, 250-450 ms) brain rhythmic activity was chosen for time-frequency analysis. 2 (group: meditators and controls) × 3 (condition: standard, target and distractor) × 4 (electrode site: Fz, FCz, Cz, and Pz) repeated measures ANOVA were conducted on the value of δ and θ respectively. All analyses were conducted via SPSS 25.0. Based on the Greenhouse–Geisser method, p-values were computed for deviation in all analyses. Simple effect analyses were conducted for multiple pairwise comparisons.

## Results

The results of the repeated measures ANOVAs using the original data for the three stimuli are presented in Table S2.

Grand average ERPs using the original data of the three stimuli for P2 and P3 at Fz and topography plots are shown in Figure S.1A. The delta and theta power at Fz is shown in Figure S.1B.

| **Table S2.** The results of the repeated measures ANOVAs using the original data for the three stimuli | | | | | | |
| --- | --- | --- | --- | --- | --- | --- |
| **Variance** | **Sum of squares** | **df** | **F** | **Sig.** | ***η²*** | **Post hoc (M±SD)** |
| P200- Amplitude |  |  |  |  |  |  |
| group | 3291.628 | 1 | 64.346 | **<0.001** | 0.668 | M (3.582±0.516)>C (0.243±0.273) |
| condition | 8.024 | 1 | 3.232 | 0.082 | 0.092 |  |
| condition × group | 0.166 | 1 | 0.067 | 0.798 | 0.002 |  |
| electrode site | 50.862 | 1 | 9.151 | **0.005** | 0.222 | FZ(3.119±0.416), FCZ(3.168±0.429), CZ(2.963±0.385)>PZ(2.132±0.268) |
| electrode site × group | 2.346 | 1 | 0.422 | 0.521 | 0.013 |  |
| electrode site × condition | 6.942 | 1 | 22.065 | **<0.001** | 0.408 | DFZ (3.348±0.468), TFZ (3.339±0.475)>SFZ (2.669±0.359); TFCZ (3.384±0.487), DFCZ (3.334±0.483)> SFCZ (2.784±0.373) |
| P300- Amplitude |  |  |  |  |  |  |
| group | 9.838 | 1 | 0.244 | 0.625 | 0.008 |  |
| condition | 92.558 | 2 | 10.546 | **<0.001** | 0.248 | T(2.378±0.463), D(1.926±0.299) > S(1.218±0.242) |
| condition × group | 51.434 | 2 | 5.861 | **0.005** | 0.155 | MT(2.703±0.574), MD(2.407±0.435)>MS(0.879±0.352) |
| electrode site | 0.591 | 3 | 0.063 | 0.979 | 0.002 |  |
| electrode site × group | 16.284 | 3 | 1.744 | 0.163 | 0.052 |  |
| electrode site × condition | 12.429 | 6 | 6.467 | **<0.001** | 0.168 | TFZ(2.666±0.551)>DFZ(1.908±0.362)>SFZ(0.953±0.362); TFCZ(2.389±0.528), DFCZ(1.964±0.355)>SFZ(1.101±0.280); TCZ(2.259±0.504), DCZ(2.118±0.334)>SCZ(1.328±0.277) |
| Delta (δ) |  |  |  |  |  |  |
| group | 1.203 | 1 | 0.69 | 0.412 | 0.021 |  |
| condition | 4.621 | 1 | 59.298 | **<0.001** | 0.649 | T(0.793±0.077), D(0.804±0.076)>S(0.542±0.051) |
| condition × group | 0.01 | 1 | 0.13 | 0.72 | 0.004 |  |
| electrode site | 7.137 | 1 | 69.435 | **<0.001** | 0.685 | FZ(0.843±0.078) > FCZ(0.810±0.067) > CZ(0.722±.070)> PZ(0.478±0.056) |
| electrode site × group | 1.147 | 1 | 11.162 | **0.002** | 0.259 | MFZ(0.978±0.114)>MFCZ(0.874±0.098)>MCZ(0.753±0.101)>MPZ(0.465±0.082); CFZ(0.708±0.107), CFCZ(0.747±0.092),CCZ(0.690±0.095)>CPZ(0.490±0.077) |
| electrode site × condition | 0.189 | 1 | 3.383 | 0.075 | 0.096 |  |
| Theta (θ) |  |  |  |  |  |  |
| group | 9.838 | 1 | 0.244 | 0.625 | 0.008 |  |
| condition | 0.677 | 1 | 12.273 | **0.001** | 0.277 | T(1.098±0.107) > D(1.005±0.101) > S(0.905±0.100) |
| condition × group | 0.249 | 1 | 4.521 | **0.041** | 0.124 | MT(1.102±0.156)>MD(1.074±0.147)>MS(0.914±0.146);CT(1.093±0.147)>CS(0.896±0.138); |
| electrode site | 22.937 | 1 | 92.546 | **<0.001** | 0.743 |  |
| electrode site × group | 0.821 | 1 | 3.311 | 0.078 | 0.094 |  |
| electrode site × condition | 0.152 | 1 | 13.999 | **0.001** | 0.304 | TFZ(1.510±0.156)>DFZ(1.281±0.119)>SFZ(1.140±0.122); DFCZ(1.167±0.106)>SFZ(1.020±0.103); TCZ(1.067±0.103)>DCZ(0.923±0.099)>SCZ(0.828±0.095); |
| **Note.** M, meditators; C, controls; S, responds in standard trials; T, responds in target trials；D, responds in distractor trials; MS, meditators in standard trials; MT, meditators in target trials；MD, meditators in distractor trials；MFZ/MFCZ/MCZ/MPZ, meditators at FZ/FCZ/CZ/PZ site; CFZ/CFCZ/CCZ/CPZ, controls at FZ/FCZ/CZ/PZ site; SFZ/SFCZ/SCZ/SPZ, standard trials in FZ/FCZ/CZ/PZ site; TFZ/TFCZ/TCZ/TPZ, target trials in FZ/FCZ/CZ/PZ site；DFZ/DFCZ/DCZ/DPZ, distractor trials in FZ/FCZ/CZ/PZ site. | | | | | | |


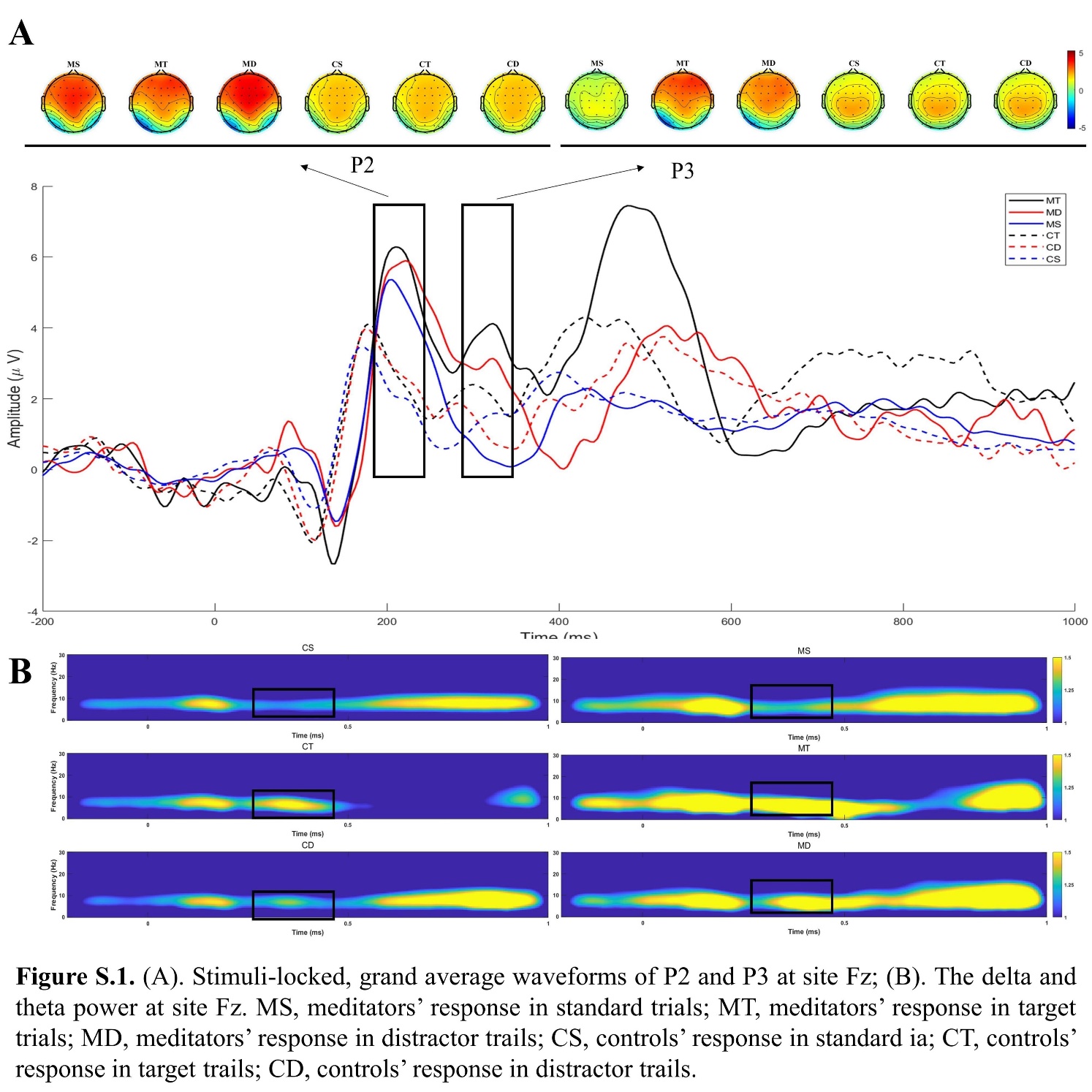


# The results of the repeated measures ANOVAs for all variances

The results of the repeated measures ANOVAs for all variances are presented in Table S3.

| Table S3. The results of the repeated measures ANOVAs for all variances | | | | | | | |
| --- | --- | --- | --- | --- | --- | --- | --- |
| Variance | Sum of squares | df | F | | Sig. | η² | Post hoc (M±SD) |
| P2 |  |  | |  |  |  |  |
| group | 130.149 | 1 | | 12.11 | **0.001** | 0.921 | M (1.629±0.290)>C (0.243±0.273) |
| condition | 3.648 | 1 | | 1.034 | 0.317 | 0.167 |  |
| condition × group | 2.653 | 1 | | 0.752 | 0.392 | 0.134 |  |
| electrode site | 37.625 | 1 | | 20.683 | **<0.001** | 0.993 | FZ(1.371±0.255)>FCZ(1.147±0.225)>CZ(0.873±0.207)>PZ(0.352±0.184) |
| electrode site × group | 7.78 | 1 | | 4.277 | **0.047** | 0.518 | MFZ(2.207±0.536)>CFZ(0.536±0.350); MFCZ(1.955±0.339)> CFCZ(0.339±0.309); MCZ(1.665±0.301)> CCZ(0.080±0.283); |
| condition × electrode site | 1.205 | 1 | | 1.807 | 0.188 | 0.256 |  |
| P3 |  |  | |  |  |  |  |
| group | 89.434 | 1 | | 1.045 | 0.314 | 0.168 |  |
| condition | 246.021 | 1 | | 13.857 | **<0.001** | 0.95 | T (3.509±0.782)>D (1.603±0.388) |
| condition × group | 55.551 | 1 | | 3.129 | 0.086 | 0.404 | MT (4.536±1.138)>MD (2.482±1.073) |
| electrode site | 87.685 | 1 | | 6.724 | **0.014** | 0.71 | FZ(1.953±0.659), FCZ(2.087±0.702)<CZ(2.760±0.607), PZ(3.424±0.470) |
| electrode site × group | 8.6 | 1 | | 0.66 | 0.423 | 0.124 |  |
| condition × electrode site | 48.89 | 1 | | 9.418 | **0.004** | 0.845 | TCZ(3.668±0.839)>DCZ(1.853±0.425); TPZ(5.173±0.625)>DPZ(1.675±0.370) |
| Delta (δ) |  |  | |  |  |  |  |
| group | 3.459 | 1 | | 6.861 | **0.013** | 0.719 | M (0.326±0.063)>C (0.100±0.059) |
| condition | 2.091 | 1 | | 7.986 | **0.008** | 0.782 | T(0.300±0.070)>D(0.125±0.028) |
| condition × group | 0.532 | 1 | | 2.033 | 0.164 | 0.282 | MT (0.458±0.101)>MD (0.143±0.096) |
| electrode site | 2.394 | 1 | | 27.866 | **<0.001** | 0.999 | FZ(0.3±0.058), FCZ(0.299±0.055)>CZ(0.198±0.045)>PZ(0.053±0.024) |
| electrode site × group | 0.715 | 1 | | 8.326 | **0.007** | 0.799 | MFZ(0.466±0.085)>CFZ(0.133±0.080);MFCZ(0.450±0.079)>CFCZ(0.148±0.075);  MCZ(0.304±0.065)>CCZ(0.093±0.061) |
| condition × electrode site | 0.344 | 1 | | 5.281 | **0.028** | 0.606 | TFZ (0.426±0.097) >DFZ (0.173±0.042); TFCZ (0.416±0.092) >DFCZ (0.182±0.036); TCZ (0.271±0.069) >DCZ (0.125±0.029) |
| Theta (θ) |  |  | |  |  |  |  |
| group | 5.003 | 1 | | 7.455 | **0.010** | 0.754 | M (0.308±0.072)>C (0.036±0.068) |
| condition | 0.782 | 1 | | 4.189 | **0.049** | 0.51 | T(0.225±0.069)>D(0.118±0.040) |
| condition × group | 0.415 | 1 | | 2.225 | 0.146 | 0.304 | MT (0.400±0.100)>MD (0.050±0.094) |
| electrode site | 3.289 | 1 | | 28.9 | **<0.001** | 0.999 | FZ(0.267±0.066), FCZ(0.280±0.057)>CZ(0.162±0.050)>PZ(-0.022±0.042) |
| electrode site × group | 0.96 | 1 | | 8.439 | **0.007** | 0.804 | MFZ(0.467±0. 096)>CFZ(0.067±0.091); MFCZ(0.456±0.083)>CFCZ(0.103±0.079);  MCZ(0.292±0.073)>CCZ(0.033±0.069) |
| condition × electrode site | 0.437 | 1 | | 4.698 | **0.038** | 0.557 | TFZ(0.363±0.098)>DFZ(0.171±0.054); TFCZ(0.369±0.087)>DFZ(0.190±0.045) |
| **Note.** M, meditators；C, controls; T, responds in target trials；D, responds in distractor trials；MT, meditators in target trials；MD, meditators in distractor trials；MFZ/MFCZ/MCZ/MPZ, meditators at FZ/FCZ/CZ/PZ site；CFZ/CFCZ/CCZ/CPZ, controls at FZ/FCZ/CZ/PZ site；TFZ/TFCZ/TCZ/TPZ, target trials in FZ/FCZ/CZ/PZ site；DFZ/DFCZ/DCZ/DPZ, distractor trials in FZ/FCZ/CZ/PZ site. | | | | | | | |
